# What is comprehensive school eye health?

**Published:** 2017-08-07

**Authors:** Hasan Minto, May Ho

**Affiliations:** 1 Director of Programmes Our Children's Vision, Brien Holden Vision Institute, Sydney NSW, Australia; 2 Education Programme Manager, Brien Holden Vision Institute, Sydney, NSW, Australia

**Education has the potential to change individuals' lives and fuel social transformation. There is a strong link between children's health, including their visual health, and the quality of their learning and achievement at school. This, in turn, affects children's future quality of life and economic productivity. School eye health programmes provide a unique opportunity to deliver comprehensive eye health services to school-going children.**

**Figure:**
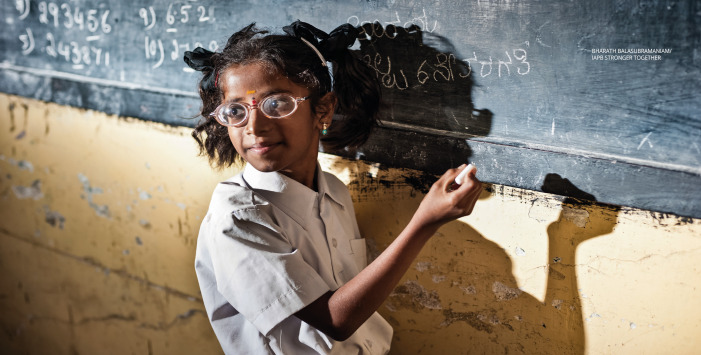
Good vision at school is essential for learning. INDIA

The World Health Organization (WHO) reports that 43% of all visual impairment is due to uncorrected refractive errors. This amounts to 122.5 million people, 12 million of whom are children.^1^

A recent study demonstrates that programmes for the detection and treatment of uncorrected refractive error (URE) among school children are highly cost effective.^2^

Comprehensive school eye health programmes are not just about URE, but can also have a positive impact on locally endemic diseases such as vitamin A deficiency or trachoma. School eye health programmes should also include identifying and referring children with other eye conditions such as strabismus or lens opacities. Health promotion and education in schools can reduce the spread of epidemic diseases such as viral conjunctivitis and reduce the risk of eye injuries. The eye health of teachers can also be addressed by detecting and managing any refractive errors, including presbyopia. The steps involved in developing a plan are shown in Figure 1.

## Children's rights, the WHO and the UN Sustainable Development Goals

The United Nations Convention on the Rights of the Child (UNCRC) recognises that children have rights of their own: the right to health, including treatment of illness and rehabilitation, a right to education and a right to an adequate standard of living. Poor eye health affects the realisation of these rights. UNCRC provides a mandate for communities, civil society and governments to come together to address child eye health.

The WHO calls for activities in prevention, treatment and rehabilitation to promote eye health in children. These activities are also enshrined in several of the United Nation's Sustainable Development Goals:

- Goal 1: Ending poverty
- Goal 3: Providing good health
- Goal 4: Providing quality education
- Goal 5: Gender equality
- Goal 8: Ensuring economic growth through the provision of good jobs.

## Challenges of current school eye health initiatives

Many school eye health initiatives are narrow in focus, do not involve ministries of health or education and are not integrated into other school health initiatives. Often, they do not provide periodic vision screening to identify new cases, nor follow up children previously identified with myopia (which can progress with age). These factors can lead to poor co-ordination, lack of ownership and affect the sustainability of programmes.

**Figure 1:**
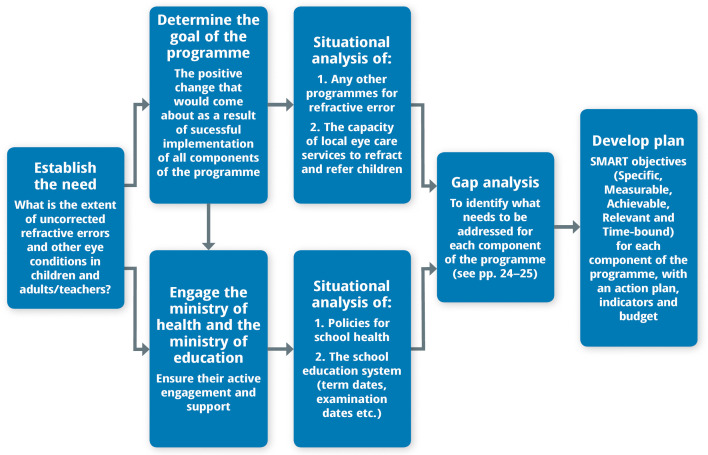
Steps in developing a comprehensive school eye health programme

There has also been a lack of standard approaches to screening, referral, prescribing, dispensing and follow-up, and most programmes do not address the eye health needs of teachers. Many of these topics are addressed in the new Standard Guidelines for Comprehensive School Eye Health Programmes.^3^

Another challenge is the inadequate monitoring and evaluation of school eye health initiatives. This can lead to inefficiencies, with poor assessment of outcomes and impact. There is evidence that a high proportion of children given spectacles do not wear them for a range of reasons, many of which could be minimised or overcome by educating parents, teachers, the children affected and their peers. Spectacle wear can be increased by dispensing spectacles only to children who really need them (see article on p. 31) and by ensuring that comfortable, cosmetically acceptable spectacles are provided free or at a minimal cost. Several of these topics are addressed in this issue.

**Figure:**
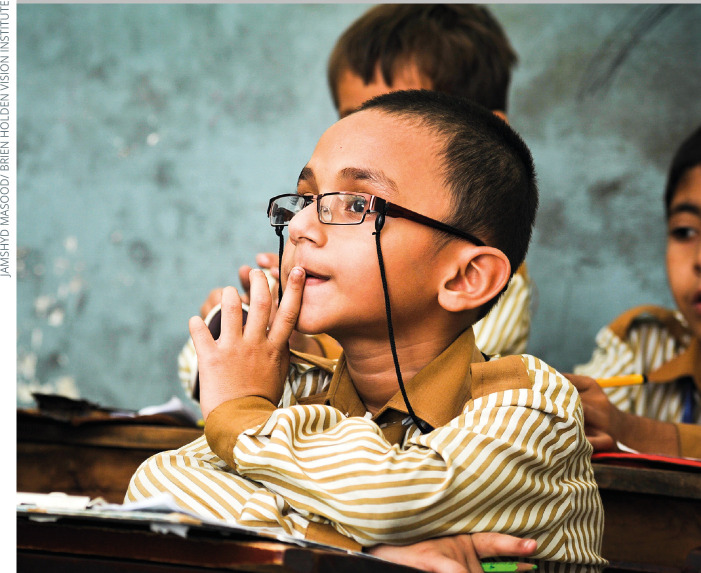
Paying attention to lessons is a lot easier if you don't have to struggle to see. PAKISTAN

## Components of a comprehensive school eye health programme

An ideal school eye health programme should be integrated into the broader school health programme and encompass the following:

### Screening, referral and treatment

- Correction of refractive error and provision of affordable, durable spectacles that are comfortable and look good.
- Identification and referral of children with other causes of visual impairment
- Identification, referral and treatment of common eye complaints in children, e.g. conjunctivitis
- Identification and referral of teachers with visual impairment
- Provision of reading spectacles for presbyopia in teachers, if required.

### Health promotion and education

- Health education to prevent locally endemic diseases in children, e.g. face washing to promote a clean face to prevent trachoma and/or good nutrition to prevent vitamin A deficiency.
- Promoting a clean, safe and healthy school environment, e.g. growing vitamin A-rich foods in a school garden, collecting water for face washing and avoiding games with sharp objects.
- Encourage children to take eye health messages home. They can act as ‘case detectors,’ identifying people in their community who need eye services. See **www.childtochild.org.uk** for more information.

The health education component of school eye health should be delivered by qualified eye care professionals, such as nurses or clinical officers, at a level that children can understand. The purpose of health education is to increase children's knowledge about the eye, how it works and what can go wrong (in simple language), and to tell them how they can keep their own eyes healthy. Ideally, this should be an integral component of the school curriculum.

**Figure 2:**
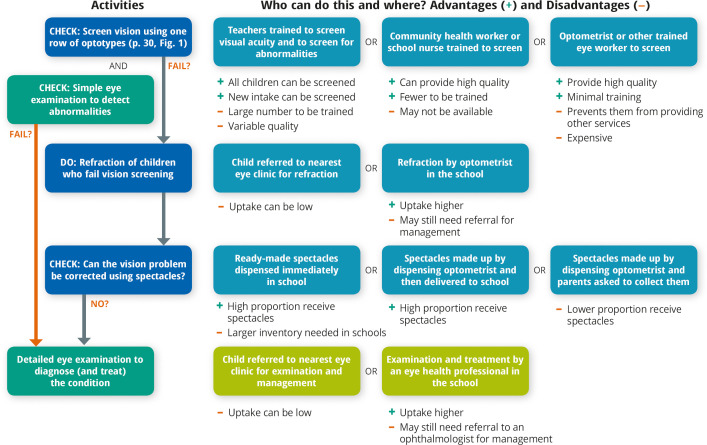
Different approaches to screening and service provision in school eye health programmes

**Figure:**
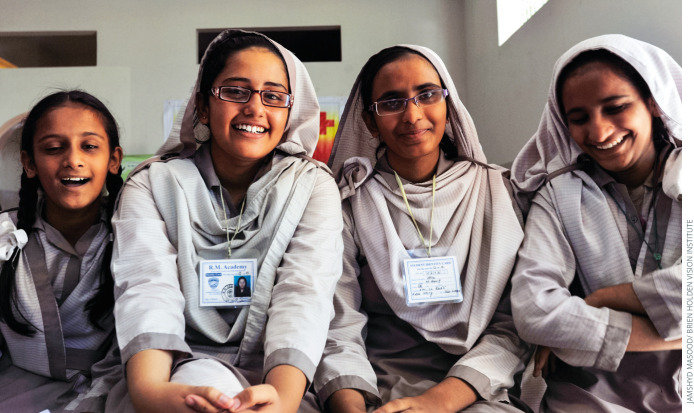
Students at a school after receiving spectacles through a school eye health programme. PAKISTAN

### Service delivery

It is desirable that the ministry of health provides financial support for programmes, including the provision of spectacles. If this is not possible, the average cost of refraction and spectacles should be kept affordable for parents.

Lack of trained eye care professionals is a major challenge in many low-income settings. The key to a successful programme is well trained and dedicated eye care personnel with clearly defined roles and responsibilities. There are several different approaches to delivering school eye health programmes; which approach is adopted largely depends on the personnel available (see Figure 2).

Ideally, refraction, prescribing and dispensing should be done in schools, as this improves children's access. If this is not possible, the next best approach is for refraction and prescribing to be done in the school, with dispensing outside: each child selects the frame they prefer in the school, and the local eye unit makes up the spectacles which are then taken back to the school.

Low power spectacles should not be provided, as they are unnecessary and will not be worn. This is a waste of resources and the programme is open to exploitation through unscrupulous prescribing. The article on p. 31 recommends prescribing based on improvement in visual acuity rather than the refractive error.

Young children do not have a well-developed bridge to their nose, and spectacle frames for children must be selected and fitted carefully. They must be cosmetically appealing, comfortable and robust enough to withstand normal wear and tear. The lenses must be able to withstand impact. Glass lenses should not be used. Plastic lenses are light, but can become scratched and should be replaced if badly scratched.

### Follow-up

The success of any programme depends on follow-up. Resources should be allocated to this and systems put in place to follow up all children who fail vision screening or who are found to have an eye condition. Follow-up may be needed after referral for refraction, to obtain spectacles, or for an eye examination at the local hospital or vision centre. Accurate and efficient record-keeping is essential, both by those who are screening and referring and those who are receiving referrals.

## New guidelines

The document Standard Guidelines for Comprehensive School Eye Health Programmes has recently been released.^3^ It provides details on how to plan, implement and monitor school eye health programmes. It is hoped that the guidelines will help to standardise school eye health programmes globally.

**Figure:**
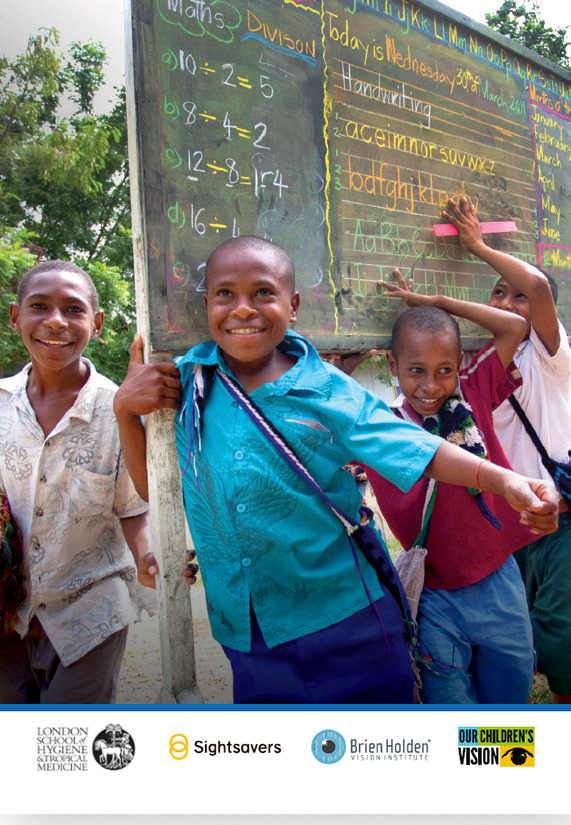
Standard Guidelines for Comprehensive School Eye Health Programs Front cover of the Standard Guidelines for Comprehensive School Eye Health Programmes.^3^
